# Cross-cultural adaptation and validation of the Romanian knee disability and osteoarthritis outcome score for joint replacement (KOOSJR)

**DOI:** 10.1186/s12891-020-3183-y

**Published:** 2020-03-07

**Authors:** Sorin Florescu, Dinu Vermesan, Horia Haragus, Jenel M. Patrascu, Bogdan Timar, Adrian Todor

**Affiliations:** 1 Department of Orthopedics and Trauma, ‘Victor Babes’ University of Medicine and Pharmacy, No 2 Eftimie Murgu Square, 300723 Timisoara, Romania; 2 Department of Functional Sciences, ‘Victor Babes’ University of Medicine and Pharmacy, No 2 Eftimie Murgu Square, Timisoara, Romania; 3 Department of Orthopedics, Traumatology and Pediatric Orthopedics, “Iuliu Hatieganu” University of Medicine and Pharmacy, No 8 Victor Babes Str, Cluj-Napoca, Romania

**Keywords:** Knee joint, Cartilage, Osteoarthritis, Arthroplasty, Knee disability and osteoarthritis outcome score for joint replacement, International knee documentation committee, Patient reported outcome measures

## Abstract

**Aim:**

To perform validation of the Romanian Knee disability and Osteoarthritis Outcome Score for Joint Replacement (KOOSJR).

**Method:**

Ninety-six patients (101 knees) with advanced osteoarthritis (OA) scheduled for total knee replacement completed Romanian translations of KOOSJR and IKDC (International Knee Documentation Committee – subjective knee form) and Euroqol EQ-5D-5 L, and the treating physician completed the original knee society score (KSS).

**Results:**

Average age was 66.4 (range 50–83) years and male to female ratio 1:3.76. There was moderate correlation between the test-retest (average 4 days) KOOSJR (*r* = 0.618, *n* = 45) and IKDC (*r* = − 0.671, *n* = 99), weak between KOOSJR and EQ-5D-5 L Index (*r* = − 0.431, *n* = 100) and VAS (r = − 0.364, n = 99) and very weak to KSS score (*r* = − 0.133, *n* = 98) and function (*r* = − 0.072, *n* = 97) For the first KOOSJR, Cronbach’s alpha was 0.816 and intraclass correlation coefficient (ICC) 0.816 (95% CI 0.755–0.866) for average measures. For the retest, Cronbach’s alpha was 0.841 (95% CI 0.760–0.903) for averages.

**Conclusion:**

The Romanian Knee disability and Osteoarthritis Outcome Score for Joint Replacement (KOOSJR) is a valid, reliable, consistent and reproducible clinical score for patients with OA requiring arthroplasty.

## Introduction

Osteoarthritis (OA) represents the degeneration of synovial joints. The knee is the most frequently involved of the large joints and its pathogenesis is not fully understood. It is accepted that OA is a disease of the entire joint, affecting the cartilage, subchondral bone and synovial tissue [1]. The prevalence of knee pain and symptomatic OA is increasing with a surge in knee arthroplasties [2].

Progression is slow and for end stages the current treatment is joint replacement. Implant survival is 95% at 10 years yet up to 20% of patients are reported to be dissatisfied. Patient-reported outcomes (PROs) are therefore an integral part of routine clinical evaluation as well as national arthroplasty registries [3]. It is recommended to use a generic tool for quality of life assessment in conjunction with a disease specific score. Knee injury and Osteoarthritis Outcome Score (KOOS) is one of the most commonly used for total knee replacement [3, 4]. A much shorter version (KOOSJR) has been proposed specifically for use in knee joint replacement. It has similar psychometric properties and its use is supported by national arthroplasty registries [3–5].

We aimed to perform validation of the Romanian translated Knee disability and Osteoarthritis Outcome Score for Joint Replacement (KOOSJR) in patients with advanced knee OA requiring joint replacement.

## Material and methods

We included 96 patients (5 bilateral) with advanced knee OA scheduled for total joint replacement, from Oct 2017 until Feb 2019. Diagnosis was made using the ACR (American College of Rheumatology) or EULAR (European League Against Rheumatism) criteria for knee osteoarthritis. Indication for arthroplasty was based on clinical and radiographic criteria (Kellgren-Lawrence grades III-IV). Bilateral cases underwent staged surgery 6 months apart. The study was conducted in accordance with the Declaration of Helsinki and the protocol was approved by the Emergency clinical county hospital ‘Pius Brinzeu’ Timisoara ‘Local ethics committee for scientific research’. All patients gave their informed consent for inclusion before they participated in the study.

The subjects completed the Romanian translations of KOOSJR and IKDC (International Knee Documentation Committee – subjective knee form) in the clinics during the preoperative evaluation [5, 6]. The treating physician (orthopedic surgeon) then completed the two parts of the original (1989) version of the knee society score (KSS1 – part1 knee score and KSS2 – part 2 function) [7]. Euroqol EQ-5D-5 L Index (converted using the UK tariff) and visual analogue scale (VAS) were used to determine the general health status [8].

The full KOOS has been previously translated into Romanian and is available on the developer’s website, yet no validation study exists [9, 10]. Equivalent questions (S6, P2, P3, P6, P9, A3 and A5) and instructions were retrieved from the full questionnaire.

Methodology and reporting agrees with the COSMIN guidelines [11]. Convergent validity was tested using Spearmans’s correlation coefficient between the tested scores. Reliability and internal consistency were determined using Cronbach’s alpha coefficient and intraclass correlation coefficient (ICC, two-way mixed effects model) [9, 10]. A subgroup of patients repeated the KOOSJR after an average of 4 days (range 2–7) for the test–retest reproducibility assessment using Spearmans’s correlation. AfFor all tests, higher values are associated with better results. Data were analyzed using SPSS v17 statistical software package (SPSS Inc., Chicago, IL, USA).

## Results

One hundred valid sets were available for processing. Average age was 66.4 (range 50–83) years and male to female ratio 21:79 (1:3.76). Forty-six subjects repeated the KOOSJR after 4 days. There were no floor or ceiling effects for both KOOSJR scores (min 0 – max 28). Raw summed scores ranged from 7 to 26 for the first and 9–27 for the second.

Twelve out of 14 consecutive patients (2 declined) were interviewed and timed at the first completion of the KOOSJR. Ten required glasses to read the questionnaire. Ten patients completed the score in an average of 2 min and 34 s and found it clear and straight forward. Four estimated that they could complete the questionnaire through mail and phone and even email or tablet with assistance from family members. There were 2 outliers, which required repeated assistance from the investigator and family members.

**Fig. 1:**
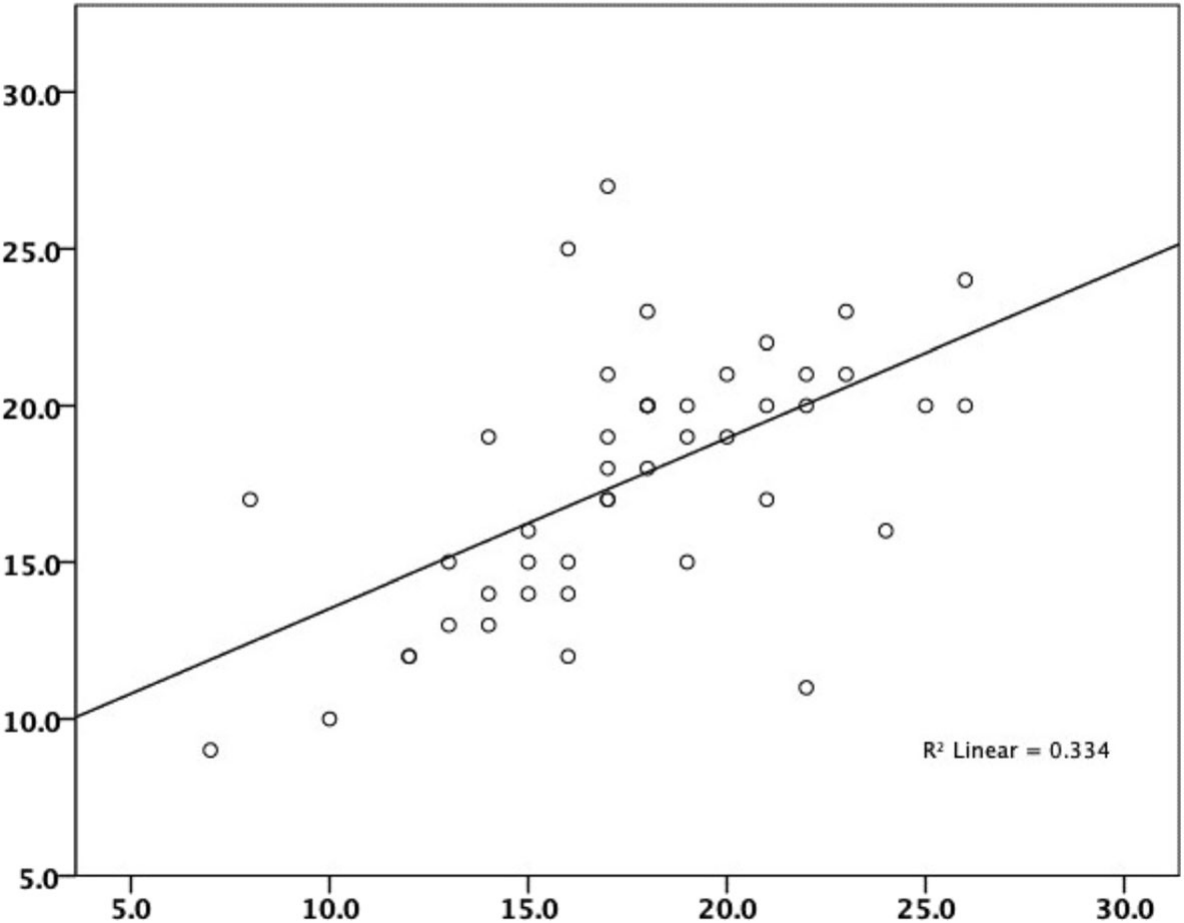
Moderate correlation between the first and repeated administration of the KOOSJR (Knee disability and Osteoarthritis Outcome Score for Joint Replacement)

**Fig. 2:**
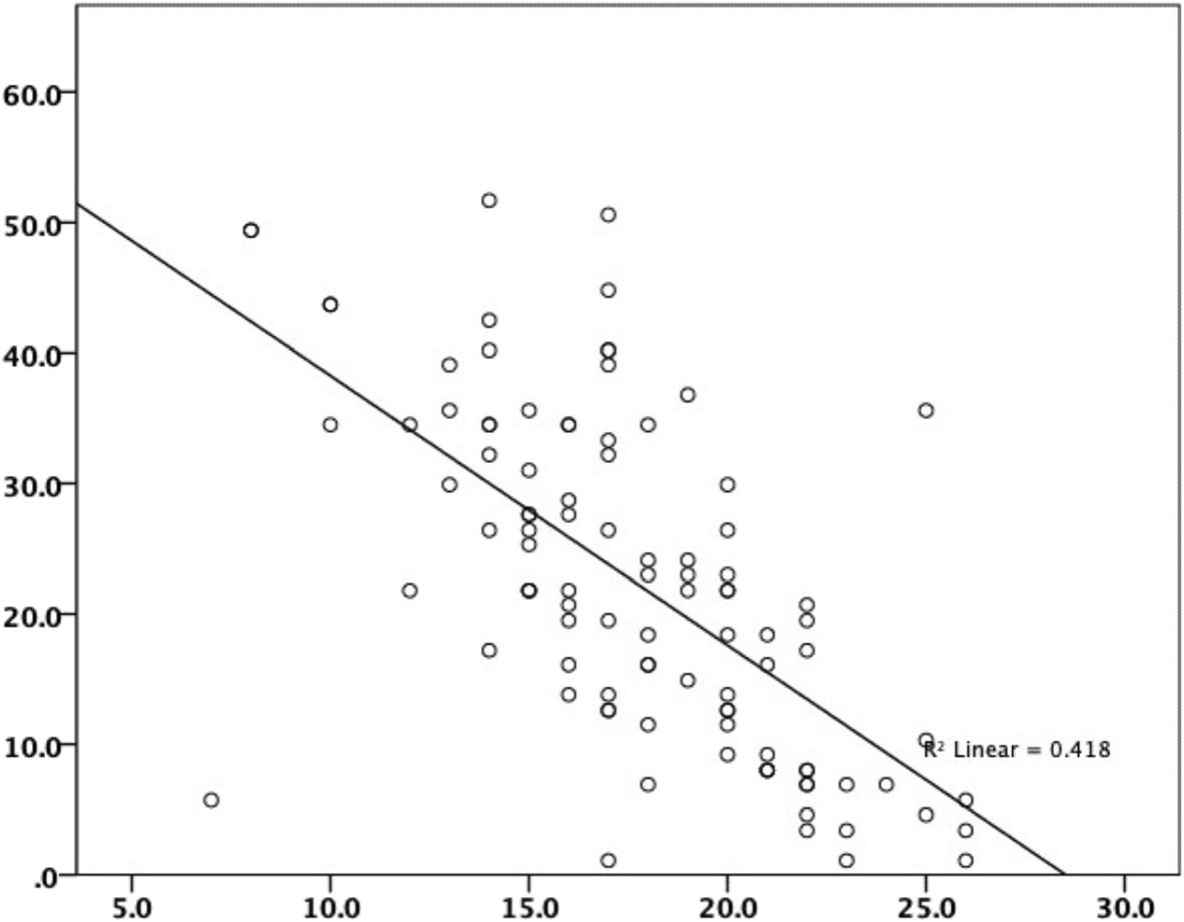
Moderate correlation between KOOSJR (Knee disability and Osteoarthritis Outcome Score for Joint Replacement) and IKDC (International Knee Documentation Committee – subjective knee form)

**Fig. 3:**
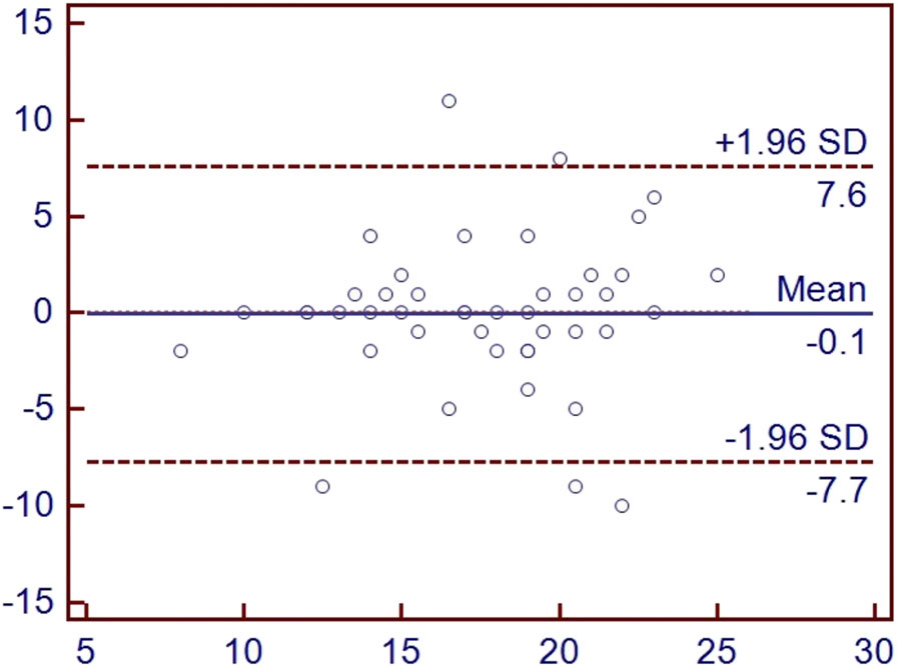
Agreement between the first and repeated administration of the KOOSJR (Knee disability and Osteoarthritis Outcome Score for Joint Replacement) presented as Bland Altman plot

There was moderate correlation between the first and repeated administration of the KOOSJR (*r* = 0.618, *n* = 45) and IKDC (*r* = − 0.671, *n* = 99), weak between KOOSJR and EQ-5D-5 L Index (*r* = − 0.431, *n* = 100) and VAS (*r* = − 0.364, n = 99) and very weak to KSS score (*r* = − 0.133, *n* = 98) and function (*r* = − 0.072, *n* = 97) (see Table 1, Figs. 1 and 2). Agreement between the first and repeated administration of the KOOSJR is presented as Bland Altman plot in Fig. 3.

**Table 1:** Correlations between the two KOOSJR (Knee disability and Osteoarthritis Outcome Score for Joint Replacement) scores, EQ-5D-5 L Index and VAS (Visual analog scale), knee society score (KSS1 – part1 knee score and KSS2 – part 2 function) and IKDC (International Knee Documentation Committee – subjective knee form)

	KOOSJR1	KOOSJR2	Index	VAS	KSS1	KSS2	IKDC
KOOSJR1	1.000	.618^b^	−.431^b^	−.364^b^	−.133	−.072	−.671^b^
	.	.000	.000	.000	.192	.485	.000
	100	45	100	99	98	97	99
KOOSJR2	.618^b^	1.000	−.563^b^	−.438^b^	.020	.131	−.484^b^
	.000	.	.000	.003	.898	.402	.001
	45	46	45	45	44	43	45
Index	−.431^b^	−.563^b^	1.000	.556^b^	.100	.255^a^	.370^b^
	.000	.000	.	.000	.330	.012	.000
	100	45	100	99	98	97	99
VAS	−.364^b^	−.438^b^	.556^b^	1.000	.229^a^	.249^a^	.377^b^
	.000	.003	.000	.	.024	.014	.000
	99	45	99	99	97	96	98
KSS1	−.133	.020	.100	.229^a^	1.000	.359^b^	.129
	.192	.898	.330	.024	.	.000	.209
	98	44	98	97	98	97	97
KSS2	−.072	.131	.255^a^	.249^a^	.359^b^	1.000	.078
	.485	.402	.012	.014	.000	.	.448
	97	43	97	96	97	97	96
IKDC	−.671^b^	−.484^b^	.370^b^	.377^b^	.129	.078	1.000
	.000	.001	.000	.000	.209	.448	.
	99	45	99	98	97	96	99

Presented as coefficient/ *p* value and number of subjects

^a^Correlation is significant at the 0.05 level (2-tailed); ^b^Correlation is significant at the 0.01 level (2-tailed)

Internal consistency was strong. For the first KOOSJR, Cronbach’s alpha was 0.816 and intraclass correlation coefficient (ICC) 0.387 for single (95% CI 0.305–0.480) and 0.816 for average (95% CI 0.755–0.866) measures respectively. For the retest, Cronbach’s alpha was 0.841 and intraclass correlation coefficient (ICC) 0.431 for single (95% CI 0.311–0.569) and 0.841 for average (95% CI 0.760–0.903) measures.

## Discussion

The Romanian translation of the KOOSJR proved valid, reliable, consistent and reproducible in patients with end stage OA undergoing total knee replacement. The Cronbach’s alpha and ICC were comparable to recently published literature regarding KOOS translations: Spanish Cronbach’s 0.78–0.93 and ICC 0.76 to 0.91; Finnish Cronbach’s 0.79–0.96 and ICC 0.73–0.86; Chinese Cronbach’s 0.76–0.97 and ICC 0.89–0.95 and Greek ICC 0.76–.89 [12–15].

A direct comparison to similar translations of KOOSJR is difficult since data is only available for the full KOOS. This later has 7 questions for symptoms, 9 for pain, 17 for activities of daily living, 5 for sports and 4 for quality of life for a total of 42 5 point Likert scale items. It is freely available, self-explanatory, comprehensive, widely used for knee injuries leading to arthritis or OA and also includes the proprietary WOMAC (Western Ontario and McMaster Universities Osteoarthritis Index) [5, 9].

Nevertheless, for routine clinical use especially in elderly, shorter scores are favored for compliance and efficiency. The 7-question standardized KOOSJR was proven to be just as reliable in patients undergoing total knee replacement. Other shortened versions of the KOOS have been proposed: the 7 question KOOS-PS (physical activity) and recently the 12 question KOOS-12 [16]. A single question – the M-SANE asked patients to rate their native or prosthetic knee on a scale from 0 to 10. It corelated strong to moderate to KOOSJR and PROMIS (Patient-Reported Outcomes Measurement Information System) physical component [17].

The PROMIS uses computer adaptive testing, where algorithms select the best questions from a larger database. It is aimed at offering a unified tool for use in different pathologies as well as integrate disease specific points to activities of daily living. Its responsiveness is comparable to KOOSJR and HOOSJR (Hip disability and Osteoarthritis Outcome Score for Joint Replacement) in patients undergoing total joint arthroplasty [18].

Our study has several limitations. Firstly, we did not use the entire KOOS and secondarily subtract the KOOSJR. We felt that the full score might have been rather cumbersome to use in current elderly population undergoing knee arthroplasty in Romania and the simplified KOOSJR was proven to offer comparable usefulness [3–5, 16]. Furthermore, we did not test responsiveness, by including a timepoint test after surgery and the translated Romanian IKDC form, the strongest comparator for validity is currently undergoing validation. The original KSS score had several limitations including high variability, acknowledged by the developer and addressed by complete revision in 2011. When our study was designed, the original KSS had been the standard of use in our clinic and the new score was not yet available free of charge. This may justify the very weak correlation found in our study between the KSS patient form and function and all other tested scores.

Knee OA is the common endpoint for a multitude of pathologies. Until present, there are no disease modifying drugs and treatment of early stages is mainly symptomatic. Total knee arthroplasty has become the mainstay for advanced disease in the elderly for many years, yet still some patients exhibit unreliable improvements. Prediction models may be one way to stratify patients at risk of poor outcomes. A group of researchers found low Oxford knee scores, poverty, increased body mass index, anxiety and depression to predict worse outcomes. In addition, there are also local factors such as impaired physical status and previous knee arthroscopy that are negative predictors of outcome. Contrarily, a fixed flexion deformity and absence of the anterior cruciate ligament were associated with postoperative improvement [19]. Machine learning algorithms are still at the beginning but show promising ability to predict which patients will achieve increased improvement after knee replacement [20].

## Conclusion

The Romanian Knee disability and Osteoarthritis Outcome Score for Joint Replacement (KOOSJR) is a valid, reliable, consistent and reproducible clinical score for patients with OA requiring arthroplasty.

## Supplementary information

**Additional file 1.** Romanian KOOSJR form.

**Additional file 2.** Raw data sets.

## Data Availability

The datasets generated and/or analyzed during the current study are not publicly available but are available from the corresponding author on reasonable request.

## Abbreviations

ACR: American College of Rheumatology
EQ-5D-5 L: (Euroqol Index) and VAS (Euroqol visual analogue scale)
EULAR: European League Against Rheumatism
ICC: Intraclass correlation coefficient
IKDC: International Knee Documentation Committee – subjective knee form
ISPOR: International Society for Pharmacoeconomics and Outcomes Research
KOOS: Knee injury and Osteoarthritis Outcome Score
KOOSJR: Knee disability and Osteoarthritis Outcome Score for Joint Replacement
KSS: Knee society score – part1 knee score and part 2 function
OA: Osteoarthritis
PROMIS: Patient-Reported Outcomes Measurement Information System
PROs: Patient-reported outcomes
WOMAC: Western Ontario and McMaster Universities Osteoarthritis Index

## References

[1] Martel-Pelletier J, Barr AJ, Cicuttini FM (2016). Osteoarthritis. Nat Rev Dis Primers.

[2] Nguyen US, Zhang Y, Zhu Y, Niu J, Zhang B, Felson DT (2011). Increasing prevalence of knee pain and symptomatic knee osteoarthritis: survey and cohort data. Ann Intern Med.

[3] Wilson I, Bohm E, Lübbeke A (2019). Orthopaedic registries with patient-reported outcome measures. EFORT Open Rev.

[4] Rolfson O, Eresian Chenok K, Bohm E (2016). Patient-reported outcome measures in arthroplasty registries. Acta Orthop.

[5] Lyman S, Lee YY, Franklin PD (2016). Validation of the KOOS, JR: a short-form knee arthroplasty outcomes survey. Clin Orthop Relat Res.

[6] Todor A, Vermesan D, Haragus H, Patrascu JM, Timar B, Cosma DI (2020). Cross-cultural adaptation and validation of the Romanian international knee documentation committee—subjective knee form. PeerJ.

[7] Orthopaedicscores website https://www.orthopaedicscore.com/scorepages/knee_society_score.html (4 Sept 2019, date last accessed).

[8] EuroQol website https://euroqol.org/eq-5d-instruments/eq-5d-5l-about/ (4 Sept 2019, date last accessed).

[9] Knee disability and Osteoarthritis Outcome Score (KOOS) website http://www.koos.nu/ (23 Feb 2020, date last accessed).

[10] Onofrei RR, Amaricai E, Petroman R, Suciu O (2019). Relative and absolute within-session reliability of the modified star excursion balance test in healthy elite athletes. PeerJ.

[11] Mokkink LB, Prinsen CAC, Bouter LM, De Vet HCW, Terwee CB (2016). The COnsensus-based standards for the selection of health measurement instruments (COSMIN) and how to select an outcome measurement instrument. Braz J Phys Ther.

[12] Lizaur-Utilla A, Miralles-Muñoz FA, Gonzalez-Parreño S, Lopez-Prats FA (2019). Validation of the Spanish version of the knee injury and osteoarthritis outcome score (KOOS) for elderly patients with Total knee replacement. J Orthop Res.

[13] Multanen J, Honkanen M, Häkkinen A, Kiviranta I (2018). Construct validity and reliability of the Finnish version of the knee injury and osteoarthritis outcome score. BMC Musculoskelet Disord.

[14] Huang CC, Chen WS, Tsai MW, Wang WT (2017). Comparing the Chinese versions of two knee-specific questionnaires (IKDC and KOOS): reliability, validity, and responsiveness. Health Qual Life Outcomes.

[15] Moutzouri M, Tsoumpos P, Billis E, Papoutsidakis A, Gliatis J (2015). Cross-cultural translation and validation of the Greek version of the knee injury and osteoarthritis outcome score (KOOS) in patients with total knee replacement. Disabil Rehabil.

[16] Gandek B, Roos EM, Franklin PD, Ware JE (2019). A 12-item short form of the knee injury and osteoarthritis outcome score (KOOS-12): tests of reliability, validity and responsiveness. Osteoarthr Cartil.

[17] Austin DC, Torchia MT, Werth PM, Lucas AP, Moschetti WE, Jevsevar DS (2019). A one-question patient-reported outcome measure is comparable to multiple-question measures in Total knee arthroplasty patients. J Arthroplast.

[18] Hung M, Saltzman CL, Greene T (2017). Evaluating instrument responsiveness in joint function: the HOOS JR, the KOOS JR, and the PROMIS PF CAT. J Orthop Res.

[19] Sanchez-Santos MT, Garriga C, Judge A (2018). Development and validation of a clinical prediction model for patient-reported pain and function after primary total knee replacement surgery. Sci Rep.

[20] Fontana MA, Lyman S, Sarker GK, Padgett DE, MacLean CH (2019). Can machine learning algorithms predict which patients will achieve minimally clinically important differences from Total joint arthroplasty?. Clin Orthop Relat Res.

