# Supplementary material for: Cross-cultural adaptation and validation of the Romanian knee disability and osteoarthritis outcome score for joint replacement (KOOSJR)
Source: BMC Musculoskelet Disord. 2020 Mar 7;21:155. doi: 10.1186/s12891-020-3183-y (PMC7060578; doi:10.1186/s12891-020-3183-y)
Supplement: Supplementary file 1 — Additional file 1. Romanian KOOSJR form. [file 12891_2020_3183_MOESM1_ESM.docx]

**KOOS, JR – CHESTIONAR DE GENUNCHI**

**INSTRUCȚIUNI**: Prin intermediul acestui chestionar sunteți invitat să evaluați starea genunchiului Dvs. Informațiile vor fi utilizate pentru a monitoriza felul în care percepeți problemele cauzate de patologia existentă la nivelul genunchiului, respectiv în ce măsură reușiți să efectuați activitățile Dvs. uzuale. Vă rugăm să răspundeți la fiecare întrebare prin bifarea căsuței corespunzătoare, câte una singură în fiecare caz. Dacă nu sunteţi sigur(ă) ce variantă să alegeţi, vă rugăm să răspundeţi cât mai aproape de adevăr.

**REDOARE**

Următoarele întrebări se referă la gradul de rigiditate („înțepenire”) a articulației genunchiului pe care l-aţi resimţit **în ultima săptămână**. Rigiditatea este o senzaţie de limitare sau încetinire a mişcării articulației.

S6. Cât de severă este senzația de rigiditate a genunchiului dvs. dimineaţa, la trezire?

| Absentă | Ușoară | Moderată | Severă | Extremă |
| --- | --- | --- | --- | --- |
| □ | □ | □ | □ | □ |

**DURERE**

Cât de intensă a fost durerea resimţită **în ultima săptămână** la nivelul genunchiului, atunci când aţi executat următoarele activităţi?

P2. Răsuciri/rotiri din genunchi;

| Absentă | Ușoară | Moderată | Severă | Extremă |
| --- | --- | --- | --- | --- |
| □ | □ | □ | □ | □ |

P3. Întinderea (extensia) completă a genunchiului;

| Absentă | Ușoară | Moderată | Severă | Extremă |
| --- | --- | --- | --- | --- |
| □ | □ | □ | □ | □ |

P6. Urcarea sau coborârea scărilor;

| Absentă | Ușoară | Moderată | Severă | Extremă |
| --- | --- | --- | --- | --- |
| □ | □ | □ | □ | □ |

P9. Statul în picioare;

| Absentă | Ușoară | Moderată | Severă | Extremă |
| --- | --- | --- | --- | --- |
| □ | □ | □ | □ | □ |

**ACTIVITĂȚI ZILNICE**

Următoarele întrebări se referă la capacitatea Dvs. de a realiza activități zilnice, de a vă deplasa și auto-îngriji. Vă rugăm să precizați dificultatea resimțită în realizarea următoarelor activități, **în ultima săptămână**, dificultate datorată patologiei de la nivelul genunchiului.

A3. Ridicarea din poziția șezând;

| Absentă | Ușoară | Moderată | Severă | Extremă |
| --- | --- | --- | --- | --- |
| □ | □ | □ | □ | □ |

A5. Aplecarea până la podea / ridicarea unui obiect;

| Absentă | Ușoară | Moderată | Severă | Extremă |
| --- | --- | --- | --- | --- |
| □ | □ | □ | □ | □ |
